# *Bifidobacterium animalis* ssp. *lactis* 420 and *Cordyceps militaris* Synergistically Modulate the Gut Microbiota by Increasing Mucin 2 Production

**DOI:** 10.3390/nu18081195

**Published:** 2026-04-10

**Authors:** Ziyang Deng, Yi Wang, Jike Shuai, Miaomiao Chen, Shuai Yang, Donghong Liu, Xingqian Ye, Shiguo Chen, Haibo Pan

**Affiliations:** 1 College of Biosystems Engineering and Food Science, Zhejiang University, 866 Yuhangtang Rd., Hangzhou 310058, China; zy019393@163.com (Z.D.); jssqwangyi@163.com (Y.W.); shuaijike1221@foxmail.com (J.S.); dhliu@zju.edu.cn (D.L.); psu@zju.edu.cn (X.Y.); 2 Innovation Center of Yangtze River Delta, Zhejiang University, Jiaxing 314102, China; 3 Tonicare Electronic Commerce (Hangzhou) Co., Ltd., Hangzhou 310016, China; chenmm@tonicare.com.cn (M.C.); yangs@tonicare.com.cn (S.Y.); 4 Zhongyuan Institute, Zhejiang University, Zhengzhou 450007, China

**Keywords:** obesity, *Bifidobacterium*, *Cordyceps militaris*, microbiome, mucin 2

## Abstract

Background: Probiotics and medicine food homology are known to offer gentle approaches to prevent obesity, although it is difficult with such approaches to satisfy consumers’ requirements to lose weight quickly. The probiotic strain *Bifidobacterium animalis* ssp. *lactis* 420 (B420) and *Cordyceps militaris* synergistically prevented obesity and related disorders in high-fat diet (HFD)-fed mice. Methods: The synergistic effects correlated with improved gut integrity, diminished systemic inflammation, and enhanced glucose homeostasis. Gut microbiota analysis revealed that the bloom of the commensal *Akkermansia muciniphila* contributed to the synergistic effects by inducing a profound shift in HFD-induced gut microbiota disorder. Results: The bloom of *A. muciniphila* was significantly correlated with a boost in mucin 2 within the colon, achieved through increased goblet cell quantity and elevated mucin 2 expression. To reveal the collaborating pathway, we found that *Cordyceps militaris* did not promote the propagation of B420 in vitro or in vivo. Moreover, heat-killed B420 could not enhance the preventive efficacy of *Cordyceps militaris* against obesity caused by the HFD. Conclusions: The metabolites of live B420 and *Cordyceps militaris*-derived metabolites in the gut microbiota collaboratively promoted the production of mucin 2. Thus, our results reveal a mechanism by which a combination of probiotics and medicine food homology enhance their therapeutic effects against obesity.

## 1. Introduction

Obesity has emerged as a critical global health concern. Extensive research has consistently demonstrated that being overweight significantly increases the risk of developing multiple chronic health ailments, including type 2 diabetes, heart-related complications, and certain types of cancer [1]. Obesity is a multifaceted condition influenced by behavioral, hereditary, and environmental determinants. Investigations in rodents and humans have confirmed the pivotal role of the gut microbiome in obesity [2]. Numerous cohort studies have validated the connection between the gut microbiome and obesity.

Among commensal bacteria in the intestinal flora, *Akkermansia muciniphila* has gained increasing attention due to its beneficial health impact [3]. Oral administration of *A. muciniphila* ameliorates obesity and related metabolic disorders, including intestinal barrier dysfunction, impaired insulin sensitivity, abnormal blood sugar regulation, and fatty liver accumulation [3]. Responses to *A. muciniphila* vary significantly due to the unique basis of individual gut microbiomes [4]. Additionally, several factors weaken the health benefits of *A. muciniphila*, such as gastric acid and pepsin, which have inactivating effects in the stomach, and the continuous flow compromising mucus adhesion in the intestine [5]. Therefore, promoting the proliferation of commensal *A. muciniphila* in the gut via dietary or nutrient intervention has potential to overcome the challenges.

The probiotic strain *Bifidobacterium animalis* ssp. *lactis* 420 (B420) has been investigated for its benefits to gut microbiota and metabolic well-being in a variety of in vitro, pre-clinical, and clinical studies, including improved gut barrier function and reduced metabolic endotoxemia [6,7,8]. Mechanistically, B420 has been shown to enhance intestinal epithelial integrity, reduce circulating lipopolysaccharide (LPS) levels, and attenuate the TLR4/NF-κB inflammatory signaling cascade, while rebalancing the obesogenic diet-induced dysbiotic gut microbiota by increasing the prevalence of lean-phenotype associated taxa such as *Akkermansia muciniphila* [6]. In a 6-month randomized controlled trial with overweight adults, B420 consumption increased the abundance of *Lactobacillus* and *Akkermansia*, and its combination with the prebiotic polydextrose further enriched *Christensenellaceae*, a taxon negatively correlated with waist-area body fat mass [7]. Specifically, subjects exhibiting greater *A. muciniphila* prevalence revealed healthier signs of obesity-related metabolism markers, including body fat distribution and triglyceride and glucose homeostasis. In the same clinical cohort, B420 alone and in combination with polydextrose significantly reduced body fat mass (by 4.0% and 4.5%, respectively), waist circumference, and energy intake compared to the placebo, with changes in serum zonulin (a marker of intestinal permeability) correlating with reductions in trunk fat mass [8]. However, while B420 alone showed modest clinical efficacy (4.0% body fat reduction and 2.4% waist circumference reduction), these effects were insufficient to meet consumer expectations, motivating the exploration of combination strategies.

*Cordyceps militaris,* a parasitic fungus, infects Lepidoptera larvae and develops into fruiting bodies. It is a cosmopolitan species with a wide geographical distribution, found not only in East Asia but also across Europe and North America. Unlike *Ophiocordyceps sinensis*, which is endemic to the Tibetan Plateau and exclusively harvested from wild populations, *Cordyceps militaris* can be efficiently cultivated on artificial substrates, making it a globally accessible functional food ingredient. The dried fruit bodies of *Cordyceps militaris* have been utilized as a folk tonic agent in traditional Chinese medicine. Numerous contemporary studies have indicated their advantageous impact on obesity and accompanying metabolic conditions, including hyperlipidemia and hyperglycemia [9,10,11,12,13]. *Cordyceps militaris* fruit bodies principally feature cordycepin, ergosterol, and polysaccharides among their bioactive constituents [9]. Both cordycepin and polysaccharides obtained from *Cordyceps militaris* were reported to ameliorate obesity via regulating endogenous metabolism and gut microbiota imbalance [9,10]. The abundance of *A. muciniphila* within the intestinal flora was obviously promoted by all of the fruit bodies of *Cordyceps militaris*, cordycepin and polysaccharides [9,10,12]. However, less than 10% of body weight loss was observed in mice after 8-week treatment with the fruit bodies of *Cordyceps militaris*, cordycepin and polysaccharides, which was not considered to be a desired effect.

In this research, our objectives were to explore whether the combination of B420 and *Cordyceps militaris* fruit bodies could produce synergistic effects against HFD-induced obesity in mice and to delve into the underlying mechanisms, giving particular attention to the roles of gut microbiota composition, intestinal barrier functionality, and mucin 2 production in the colon.

## 2. Materials and Methods

### 2.1. Animal Experiments

C57Bl/6J male mice aged 8 weeks (Beijing Vital River Laboratory Animal Technology Co., Ltd., Beijing, China) were confined within a regulated environment (12 h daylight phase, illumination terminated at 18:00 h) with unrestricted access to food and water in the animal housing facility of Dr. Can Bioscience Inc. (Hangzhou, China). After 2 weeks of acclimatization on a normal-chow diet, mice were randomly divided into five groups (n = 6, N = 30): chow diet (Chow), HFD control (HFD), treatment with 2 × 10^9^ cfu/kg/day of B420 (B420), treatment with 50 mg/kg/day of *Cordyceps militaris* powder (CM) and combined treatment of B420 and *Cordyceps militaris* powder (B420+CM). All treatments (B420, CM, and B420+CM) were administered daily by oral gavage. Treatment began when the HFD diet was introduced throughout the 8 weeks. Weekly body weight measurements were recorded. At week 8, animals received anesthesia in isoflurane-saturated enclosures and were subsequently euthanized via the cardiac puncture method. Blood samples were ingathered in tubes and subjected to centrifugation to isolate serum from cellular components. Visceral fat pads were meticulously gleaned along with the liver and intestines. Experiments on animals were performed in adherence to a predetermined protocol that had been authorized by the Administrative Panel on Laboratory Animal Care of Dr. Can Bioscience Inc. (approval No. DRK-202504079821, approval date: 7 April 2025).

### 2.2. Oral Glucose Tolerance Test and Insulin Tolerance Test

Mice underwent an 8 h fast before receiving a 10% (*w*/*v*) glucose solution via intragastric gavage at a dosage of 1 g/kg for the oral glucose tolerance test. For the insulin tolerance test, mice underwent 6 h of fasting and were injected intraperitoneally with insulin (0.5 IU/kg). Blood glucose concentrations were determined via tail vein blood using Accu-Chek blood glucose meters (Roche, Shanghai, China). The total area under the curve (AUC) was calculated based on the trapezoidal method.

### 2.3. Analysis of Serum Inflammatory Cytokines with ELISA

The serum levels of tumor necrosis factor-α (TNF-α) and interleukin-1β (IL-1β) were determined using ELISA kits (Thermo Fisher Scientific, Shanghai, China) according to the manufacturer’s instructions.

### 2.4. Gut Permeability Assays

Assays for gut permeability involved administering 0.4 mg/g body weight of fluorescein-isothiocyanate (FITC)-dextran (4 kDa, Sigma, Ronkonkoma, NY, USA) to overnight-fasted mice via oral administration. Whole blood was ingathered via cardiac puncture. Serum was mixed with PBS in a 1:1 ratio prior to placement in a 96-well flat-bottom plate. Fluorescence detection occurred at 485/525 nm using a spectrophoto fluorometer (Synergy HT, BioTek, Winooski, VT, USA). Sera spiked with FITC-dextran at different concentrations were utilized to establish the standards.

### 2.5. RNA Extraction and Quantitative Real-Time PCR Analysis

RNA from the proximal colon tissues and cells was isolated using the Tri Reagent Kit (Sigma, USA) as per the manufacturer’s instructions. An equivalent quantity of RNA was converted into cDNA with the aid of the Quant II fast RT kit (Tools, Taipei, Taiwan). cDNA analysis involved quantitative real-time PCR using the KAPA SYBR FAST Universal 2× qPCR Master Mix (Kapa Biosystems, Wilmington, MA, USA). The expressions of zonula occludens-1, claudin-1, mucin 2 and glyceraldehyde-3-phosphate dehydrogenase were evaluated via qRT-PCR. The transcripts’ relative quantities for the target genes were assessed per cDNA sample post-normalization with reference to GAPDH. Data were analyzed via the 2^−ΔΔCT^ protocol. qRT-PCR primer sequences are shown in Table S1.

### 2.6. Bacterial Quantification by qPCR

The presence of *A. muciniphila* and B420 in the feces was assessed by qPCR as outlined previously [14]. Briefly, copy numbers of *A. muciniphila* and B420 per ng of fecal DNA were obtained based on the Ct values and calculated using standard curves designed for each taxon. Primers were designed in silico and tested by Primer3-BLAST (v2.5.0) analysis; their sequences are available in Table S1.

### 2.7. Immunohistochemistry Staining

Immunohistochemistry staining was conducted on paraffin-embedded sections (4 μm) of colon utilizing the Benchmark XT slide staining system coupled with the OptiView DAB IHC Detection Kit (Ventana Medical Systems, Tucson, AZ, USA). Sections were hybridized with rabbit-anti-mouse mucin 2 (Thermos fisher, Shanghai, China). Cell nuclei were counterstained using 4′,6-diamidino-2-phenylindole (DAPI).

### 2.8. Culture of B420 with Cordyceps militaris

B420 was incubated at 37 °C within a Coy Anaerobic Model A chamber, utilizing a gas blend of 10% carbon dioxide, 5% hydrogen and 85% nitrogen. An anaerobic gas infuser was utilized to sustain a hydrogen concentration of 3.3%. Culture media and plasticware were stored within an anaerobic environment for a minimum of 24 h prior to application. B420 was cultured in BHI media, of which the compositions are detailed in Table S2. Carbon source-free BHI medium was crafted by tweaking a typical BHI recipe by removing all carbohydrate elements while keeping the essential peptones, tissue infusions, and inorganic salts intact. Sterile powder of *Cordyceps militaris* was added at a dose of 8.0 mg/mL.

### 2.9. Culture of Gut Microbiota from HFD-Fed Mice with B420 and Cordyceps militaris

Fecal samples were collected and transferred to an anaerobic workstation. The fecal samples were suspended in PBS at a 1:10 (*w*/*v*) ratio. The gut microbiota was collected by filtering with a stainer and was centrifuged at 4 °C, 8000 rpm for 5 min. Waste supernatant was removed at the anaerobic workstation, while the pellets were reconstituted in the same volume of BHI media. B420 and *Cordyceps militaris* powder were suspended in BHI media at doses of 1 × 10^7^ cfu/mL and 16.0 mg/mL. The suspensions of gut microbiota and B420 or *Cordyceps militaris* powder were mixed at a 1:1 (*v*/*v*) ratio and incubated for 12 h. After incubation, each bacterial culture underwent centrifugation at 8000 rpm, 4 °C for an interval of 5 min. The supernatants were collected for cell study.

### 2.10. Cell Culture and Assessment

The HT29-MTX-E12 cells were maintained in DMEM enriched with 10% FBS and an antibiotic cocktail of 100 units/mL penicillin–streptomycin. These cells were cultivated at 37 °C within a humidified chamber supplying 5% CO_2_ to create an optimal growth environment. HT29-MTX-E12 cells were plated at a concentration of 3 × 10^5^ cells/cm^2^. For investigating the effect of the metabolites of live B420 and *Cordyceps militaris*-derived metabolites of gut microbiota on HT29-MTX-E12 cells, HT29-MTX-E12 cells were incubated with 10% PBS, 5% of each supernatant and 5% PBS or 10% of supernatants (1:1, *v*/*v*). Assessment of cellular viability was conducted via a CCK-8 cell viability assay kit (Jiancheng Bioengineering Institute, Nanjing, China) according to the manufacturer’s instructions. Alcian blue staining was performed as previously described with a minor modification [15]. Briefly, cells were fixed with ice-cold ethanol containing 5% acetic acid for 20 min and then stained with Alcian blue 8GX for 10 min at ambient temperature. Cells were rinsed with PBS and observed with an Olympus IX51 microscope (Tokyo, Japan).

### 2.11. Statistical Analysis

Statistical analyses were conducted with GraphPad Prism V10.6.1 (GraphPad Software, San Diego, CA, USA). Data are presented as means ± standard deviations (SDs). Differences assessments of the two groups were conducted via unpaired, two-tailed Student’s *t*-tests. Multiple comparisons were performed by one-way analysis of variance (ANOVA) followed by Turkey’s test. Results were considered to be statistically significant at *p* < 0.05.

## 3. Results and Discussion

### 3.1. B420 and Cordyceps militaris Synergistically Reduce Body Weight in HFD-Fed Mice

**Figure 1:**
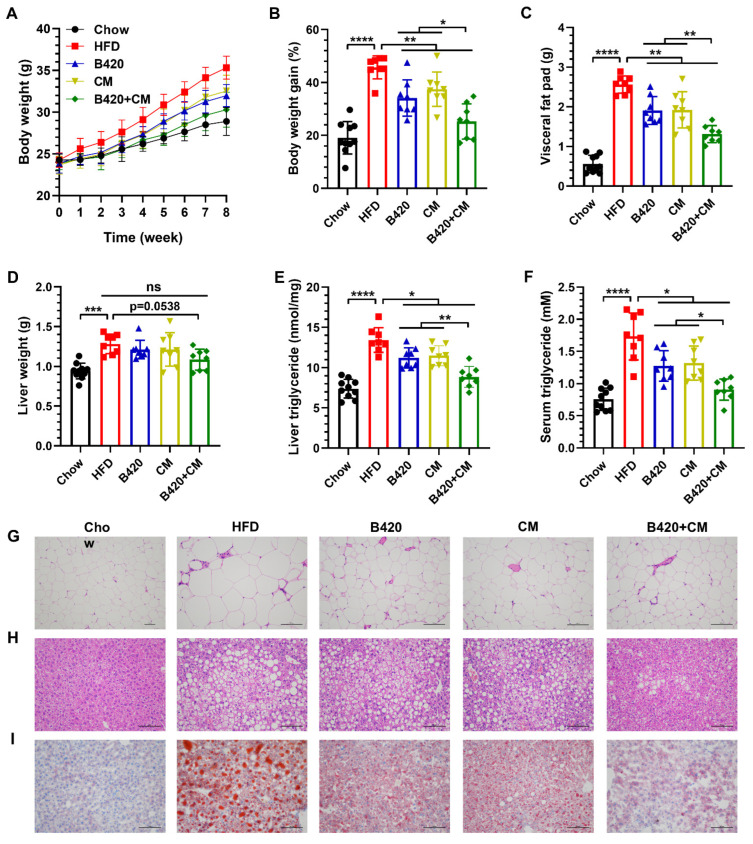
B420 and *Cordyceps militaris* synergistically prevented HFD-induced obesity. (**A**) Body weight was monitored across the 8-week period. (**B**) Body weight gain, (**C**) visceral fat pad weight, (**D**) liver weight, (**E**) liver triglycerides and (**F**) serum triglycerides were measured after the 8-week treatment. Representative images of H&E-stained (**G**) visceral adipose tissues and (**H**) liver and (**I**) oil red o-stained liver. Scale bar, 50 µm. * *p* < 0.05; ** *p* < 0.01; *** *p* < 0.001; **** *p* < 0.0001; ns, not significant.

To investigate the enhanced effects of B420 and *Cordyceps militaris* on body weight, mice were fed an HFD for 8 weeks. When contrasted to the chow diet, mice fed an HFD showed significantly elevated body weight gain, visceral fat mass, liver weight, liver triglyceride content and serum triglyceride content (Figure 1A–F). Treatment with B420, *Cordyceps militaris* and the combination significantly reduced these obesity traits. Specifically, B420, *Cordyceps militaris* and the combination reduced body weight gain by 11.6%, 8.2% and 20.4% compared with the HFD (Figure 1B), indicating the synergistic effects of B420 and *Cordyceps militaris* against HFD-induced obesity. A similar effect was observed for visceral fat mass (Figure 1C), which was confirmed via reduced adipocyte hypertrophy as analyzed by H&E staining (Figure 1G). Although the liver weight was not reduced significantly, a moderate synergistic effect was observed (*p* = 0.0538) (Figure 1D). Notably, the content of liver triglycerides displayed a synergistic effect similar to body weight gain and visceral fat mass (Figure 1E), which was accompanied by reduced liver cell hypertrophy and accumulation of lipid droplets as analyzed by H&E staining and oil red o staining (Figure 1H,I). Furthermore, B420 and *Cordyceps militaris* synergistically inhibited the increase in serum triglycerides induced by the HFD (Figure 1F).

These anti-obesity outcomes are consistent with, yet extend beyond, findings from previous individual studies on B420 and *Cordyceps militaris* [6,7,8,13,16]. Both B420 and *Cordyceps militaris* alone typically achieved less than a 12% reduction in body weight gain in rodent models, which aligns with the modest effects observed in our single-agent treatment groups. Notably, the 20.4% reduction in body weight gain achieved by the combined treatment in the present study represents a substantial improvement over either agent alone and approaches the efficacy range of interventions. In addition, the concurrent reductions in visceral fat mass, liver triglycerides, and serum triglycerides further suggest that the combined treatment exerts coordinated effects on multiple lipid metabolic pathways. The marked reduction in hepatic triglyceride content is particularly noteworthy, as hepatic steatosis is a recognized hallmark of metabolic syndrome and a key driver of systemic metabolic dysfunction. Taken together, these results indicate that B420 and *Cordyceps militaris* synergistically prevented HFD-induced obesity.

### 3.2. B420 and Cordyceps militaris Synergistically Improve Glucose Homeostasis in HFD-Fed Mice

**Figure 2:**
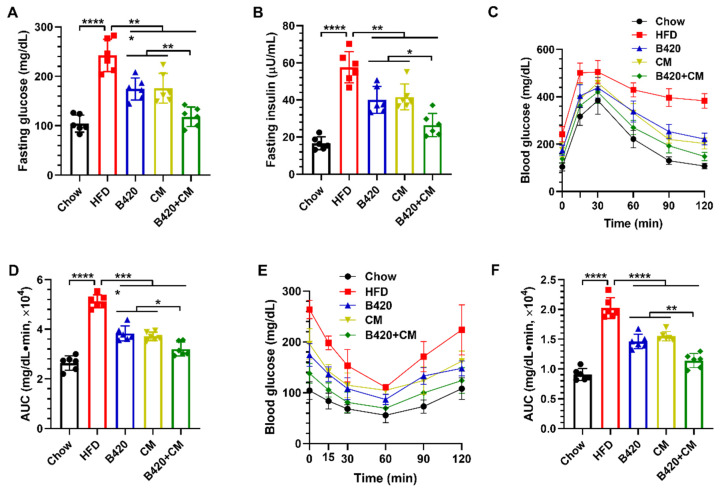
B420 and *Cordyceps militaris* synergistically improve glucose homeostasis in HFD-fed mice. (**A**) Fasting glucose and (**B**) fasting insulin. (**C**) The oral glucose tolerance test (blood glucose levels after oral glucose challenge) and (**D**) the corresponding areas under the curve (AUCs). (**E**) The insulin tolerance test (blood glucose levels after intraperitoneal insulin injection) and (**F**) the corresponding AUCs. * *p* < 0.05; ** *p* < 0.01; *** *p* < 0.001; **** *p* < 0.0001.

Obesity is strongly correlated with numerous metabolic disturbances, including type 2 diabetes, clinically evidenced by hyperglycemia and insulin resistance [17]. Weight loss helps to improve obesity-related hyperglycemia and insulin resistance [18]. To verify the synergistic prevention of B420 and *Cordyceps militaris* against obesity-enhanced glucose homeostasis in mice fed a high-fat diet, initial measurements of fasting glucose and insulin levels were conducted. Treatment with B420, *Cordyceps militaris* and the combination reduced fasting glucose and fasting insulin compared with the HFD (Figure 2A,B). Lower levels of fasting glucose and fasting insulin were observed after the combined treatment than after treatment with B420 or *Cordyceps militaris* alone. To test the effect of B420, *Cordyceps militaris* and the combination regarding glucose tolerance and insulin responsiveness, the oral glucose tolerance test and the insulin tolerance test were carried out. Glucose tolerance and insulin sensitivity were improved by B420, *Cordyceps militaris* and the combination as assessed with the oral glucose tolerance test (Figure 2C,D) and the insulin tolerance test (Figure 2E,F). The difference in the AUCs demonstrates that the combined treatment produced enhanced glucose clearance and insulin sensitivity. The improvement in glucose homeostasis observed in the study is consistent with the established metabolic benefits of both B420 and *Cordyceps militaris* reported in previous studies [6,7].

It is worth noting that the relationship between obesity, gut microbiota dysbiosis, and insulin resistance forms a vicious cycle. Breaking this cycle at multiple nodes simultaneously may explain why the combined treatment produced more pronounced improvements compared to either agent alone. The synbiotic approach combining B420 with the dietary fiber polydextrose was previously shown to reduce serum zonulin (a marker of intestinal permeability) in parallel with reduced abdominal adiposity in overweight adults [7]. Our findings extend this concept by demonstrating that pairing B420 with *Cordyceps militaris* may offer advantages beyond conventional synbiotic formulations. Specifically, whereas dietary fibers primarily serve as fermentable substrates for gut bacteria, *Cordyceps militaris* provides both prebiotic-like polysaccharides and pharmacologically active compounds such as cordycepin and ergosterol, potentially engaging both microbiota-dependent and microbiota-independent pathways to improve metabolic outcomes. These findings demonstrate that the prophylactic impact of B420 and *Cordyceps militaris* on HFD-induced obesity resulted in synergistically enhanced glucose homeostasis.

### 3.3. B420 and Cordyceps Militaris Synergistically Ameliorate Inflammation and Prevent Leaky Gut in HFD-Fed Mice

**Figure 3:**
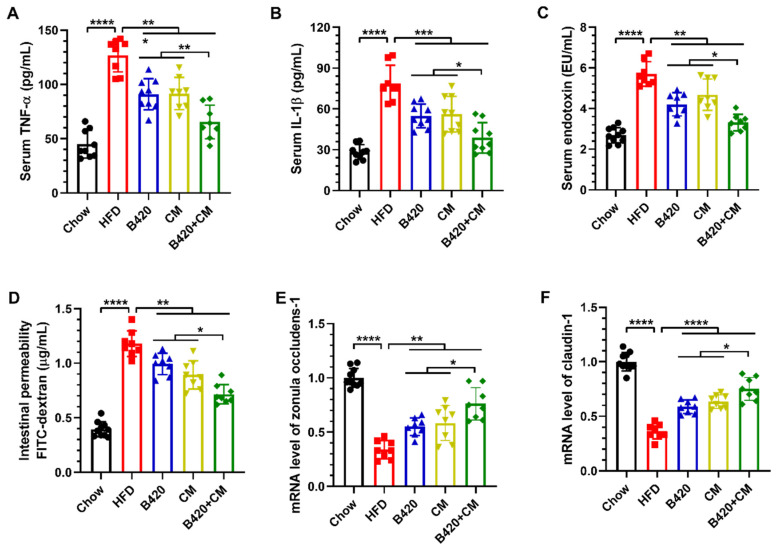
B420 and *Cordyceps militaris* synergistically ameliorate inflammation and prevent leaky gut in HFD-fed mice. Serum expression levels of (**A**) tumor necrosis factor-alpha (TNF-α), (**B**) interleukin (IL)-1β and (**C**) endotoxin. (**D**) Intestinal permeability was measured with 4 kDa FITC-labeled dextran. The expressions of (**E**) zonula occludens-1 and (**F**) claudin-1 in the proximal colon were examined using qRT-PCR. * *p* < 0.05; ** *p* < 0.01; *** *p* < 0.001; **** *p* < 0.0001.

It is known that HFD-induced obesity has been associated with disrupted gut barrier integrity and increased intestinal permeability [19]. The permeable intestinal lining leads to the translocation of bacterial metabolic endotoxemia from the intestine into the blood and subsequently triggers inflammation and insulin resistance [20]. As the synergistic prevention of B420 and *Cordyceps militaris* against HFD-induced insulin resistance was observed, we next measured their effects on inflammation and intestinal permeability. To test the synergistic effects of B420 and *Cordyceps militaris* on inflammation in HFD-fed mice, levels of the pro-inflammatory cytokines TNF-α and IL-1β in the serum were assessed using an ELISA kit. Treatment with B420, *Cordyceps militaris* and the combination reduced serum levels of TNF-α and IL-1β, with the combined treatment showing lower levels than either singular treatment (Figure 3A,B). B420 and *Cordyceps militaris* synergistically ameliorated inflammation induced by the HFD. Furthermore, treatment with B420, *Cordyceps militaris* and the combination dramatically decreased serum levels of endotoxin (Figure 3C) and intestinal permeability as evaluated with 4 kDa FITC-labeled dextran (Figure 3D) in HFD-fed mice. Notably, consistent with the effects described above on inflammation, the combined treatment ameliorated the intestinal permeability induced by the HFD more than B420 or *Cordyceps militaris* alone. These observations were accompanied by elevated mRNA expressions of colonic tight junction proteins zonula occludens-1 and claudin-1 (Figure 3E,F). Taken together, B420 and *Cordyceps militaris* synergistically prevented obesity-induced leaky gut and ameliorated inflammation in HFD-fed mice.

### 3.4. B420 and Cordyceps militaris Synergistically Prevent Obesity-Driven Dysbiosis in HFD-Fed Mice

**Figure 4:**
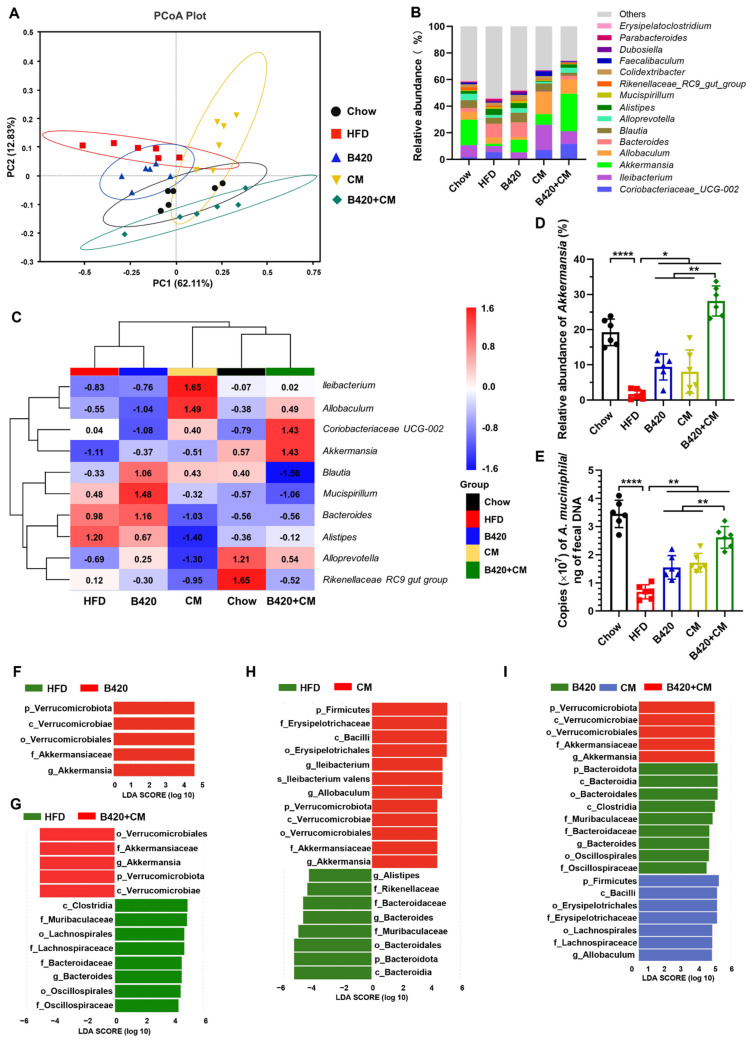
B420 and *Cordyceps militaris* synergistically prevent obesity-driven dysbiosis in HFD-fed mice. (**A**) Principal coordinate analysis (PCoA) on unweighted UniFrac distance matrix. (**B**) Relative abundance of taxa at the genus level. (**C**) Heat map showing the levels of the top 10 most abundant genus taxa. (**D**) The relative abundance of genus *Akkermansia*. (**E**) The qPCR quantification of *A. muciniphila* in feces. Linear discriminant analysis (LDA) effect size (LEfSe) was employed to delve into the most specific taxonomic level, pinpointing the classifications that most effectively distinguish the gut microbiome of (**F**) HFD vs. B420, (**G**) HFD vs. B420+CM, (**H**) HFD vs. CM, and (**I**) B420 vs. CM vs. B420+CM. * *p* < 0.05; ** *p* < 0.01; **** *p* < 0.0001.

The gut microbiota fosters the progression of diet-induced obesity and metabolic disturbances through the promotion of low-grade inflammation from bacterial components like endotoxin, increases in energy harvesting from food, and disruption of intestinal barrier integrity [21]. Gut microbiota dysbiosis can lead to increased intestinal permeability, enabling bacterial poisons to migrate into the circulatory system and triggering an immune reaction that exacerbates metabolic derangement [22]. Given that B420 and *Cordyceps militaris* synergistically decreased serum endotoxemia and gut permeability, we subsequently investigated whether the advantageous effects were mediated by the gut microbiota via 16S rRNA gene sequencing. Principal coordinate analysis demonstrated that 8 weeks of HFD feeding significantly altered the intestinal microbiome composition (Figure 4A,B). All the treatments promoted the development of different gut microbial communities. B420- and *Cordyceps militaris*-treated mice partially clustered separately among HFD-fed mice samples, while the combined treatment formed a cluster that was distinct from that of HFD-fed mice. This demonstrates that the combined treatment produced a more pronounced impact on the gut microbiome’s structural composition in HFD-fed mice than did B420 or *Cordyceps militaris* alone.

Next, to find out which bacterial genera contributed to the significant impact on the composition of the intestinal microbial ecosystem, the specifically defined comparisons were depicted in a hierarchical clustering heat map representing the z-score of averaged taxonomic abundance. The top 10 most abundant genus taxa were displayed (Figure 4C). The HFD-induced changes in the *lleibacterium*, *Akkermansia* and *Alistipes* genera were restored by all the treatments. Notably, a more marked alteration in the combined treatment group than in either the B420 or *Cordyceps militaris* group was observed in the *Akkermansia* genus (Figure 4D). The relative abundance of sequences assigned to *Akkermansia* ranged from 1.7% to 28.1%. The relative abundances of *Akkermansia* were increased to 9.4%, 8.0% and 28.1% after treatment with B420, *Cordyceps militaris* and the combination. The same tendency towards expansion of *Akkermansia* was observed with the LEfSe approach, which ranked *Akkermansia* as the only common feature discriminating fecal bacterial communities of treated mice from that of HFD control mice (Figure 4F–I). *Akkermansia* is a genus in the phylum *Verrucomicrobiota* with the type species *A. muciniphila*. Numerous animal and human studies have demonstrated that supplementation with *A. muciniphila* ameliorates obesity and associated disorders, including insulin resistance, glucose intolerance and intestinal barrier dysfunction [3,23,24,25]. In consideration of the tendency towards the abundance of genus *Akkermansia* after treatment with B420, *Cordyceps militaris* and the combination, we quantified *A. muciniphila* by qPCR, and the same changes in its copies were observed (Figure 4E). Thus, B420 and *Cordyceps militaris* synergistically prevent HFD-driven dysbiosis in mice. Notably, *A. muciniphila* made a remarkable contribution to the observed distinctions in the gut microbial community compositions in HFD-fed and treated mice.

### 3.5. B420 and Cordyceps militaris Synergistically Increase the Number of Goblet Cells and Promote the Expression of Mucin 2 in the Colon of HFD-Fed Mice

*A. muciniphila* dwells in the outer mucus layer of the colon and utilizes goblet cell-produced mucin 2 as its primary food source [26,27]. Despite degrading mucin, *A. muciniphila* increases the number of goblet cells and stimulates goblet cells to produce more mucin 2, which helps to preserve the thickness and structural soundness of the mucus barrier [28,29]. By increasing the number of goblet cells and promoting mucus layer regeneration, *A. muciniphila* enhances intestinal barrier function, leading to reduced permeability and improved protection against inflammation [30].

**Figure 5:**
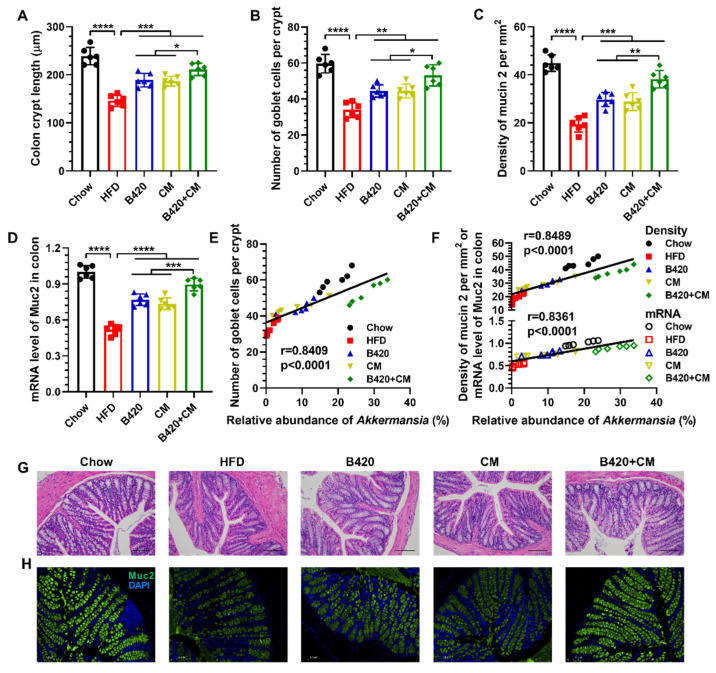
B420 and *Cordyceps militaris* synergistically increase the number of goblet cells and promote the expression of mucin 2 in the colon of HFD-fed mice. (**A**) Colon crypt length was measured based on H&E staining. (**B**) The amount of mucin-producing goblet cells per crypt and (**C**) the fluorescence density of mucin 2 per mm^2^ were measured based on immunostaining with mucin 2 and DAPI. (**D**) The expression of mucin 2 in the proximal colon was examined using qRT-PCR. The Pearson’s correlation between the relative abundance of *Akkermansia* and (**E**) the number of goblet cells, (**F**) the density of mucin 2 and the mRNA level of mucin 2. Representative images of (**G**) H&E-stained and (**H**) immune-stained colon. Scale bar, 50 µm. * *p* < 0.05; ** *p* < 0.01; *** *p* < 0.001; **** *p* < 0.0001.

In consideration of the observed changes in the copies of *A. muciniphila* after treatment with B420, *Cordyceps militaris* and the combination, the proximal colons of mice from the different treatment groups were first analyzed with H&E staining and immunostaining. We found that the HFD induced intestinal atrophy and reduced crypt length (Figure 5A,G), consistent with a marked loss of colon length and mass [31]. The reduction in crypt length was partly restored by the treatments, with the combination exhibiting the best restoration. Such promotion of colon crypt length associated with the elevated number of mucin-producing goblet cells per crypt, as measured by immunostaining with mucin 2 (Figure 5B,H). Furthermore, we evaluated their effect on the production of mucin 2 by comparing the fluorescence density of mucin 2 per mm [2]. The production of mucin 2 was synergistically promoted by B420 and *Cordyceps militaris* (Figure 5C), in accordance with the mRNA levels of mucin 2 in the proximal colon (Figure 5D). In addition, Pearson correlation analysis showed that the relative abundance of *Akkermansia* was positively correlated with the number of goblet cells per crypt (Figure 5E, r = 0.8409), the density of mucin 2 per mm [2] (r = 0.8489) and the mRNA level of mucin 2 (r = 0.8361) (Figure 5F) in the colon. Together, these results indicate that *A. muciniphila* contributed to the synergistic effect of B420 and *Cordyceps militaris* on the production of mucin 2 in the colon in HFD-fed mice.

### 3.6. Live B420, Rather than Dead B420, Collaborated with Cordyceps militaris to Produce the Synergistic Effect

**Figure 6:**
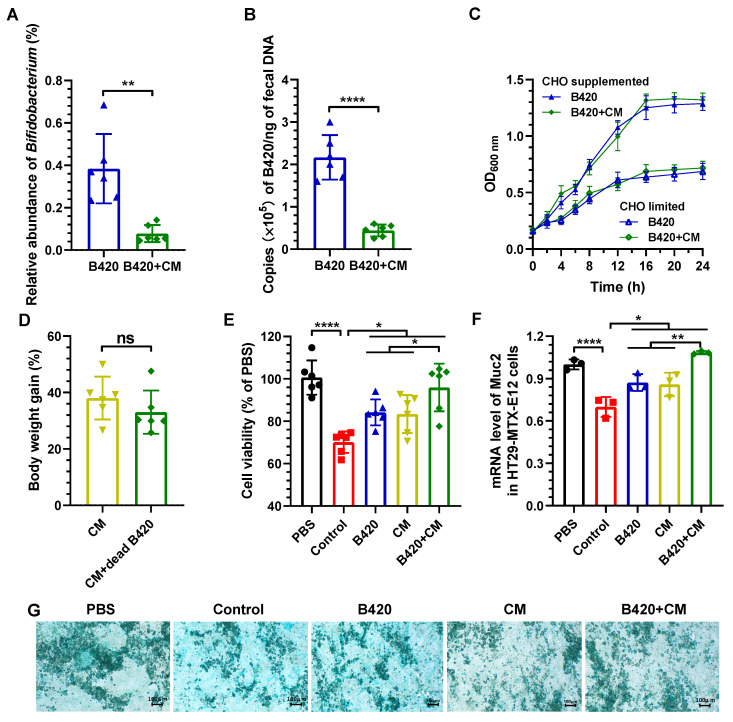
B420 and *Cordyceps militaris* synergistically increase the number of goblet cells and promote the expression of mucin 2 in the colon of HFD-fed mice. (**A**) The relative abundance of genus *Bifidobacterium*. (**B**) The qPCR quantification of B420 in feces. (**C**) The proliferation of B420 in *Cordyceps militaris*-supplemented culture medium compared to controls in the presence/absence carbohydrates. (**D**) Body weight gain was measured after the 8-week treatment with dead B420 and *Cordyceps militaris*. The effects of the metabolites of live B420 and *Cordyceps militaris* on (**E**) cell viability and (**F**) mRNA mucin 2 expression of HT29-MTX-E12 cells. (**G**) HT29-MTX-E12 cells stained with Alcian blue. Scale bar, 100 µm. * *p* < 0.05; ** *p* < 0.01; **** *p* < 0.0001; ns, not significant.

B420 and *Cordyceps militaris* were reported to multiply the relative abundance of *A. muciniphila*. In the current research, a synergistic effect of B420 and *Cordyceps militaris* on *A. muciniphila* was observed. The mechanism underlying the synergistic effect on the production of mucin 2 and subsequent promotion of *A. muciniphila* is not clear. We hypothesize that *Cordyceps militaris* could promote the proliferation of B420. Interestingly, both the relative abundance of genus *Bifidobacterium* and copies of B420 in the feces were significantly decreased by *Cordyceps militaris* as analyzed by 16S rRNA sequencing and qPCR (Figure 6A,B). It seems that *Cordyceps militaris* inhibited the proliferation of B420 in the colon. To verify the efficacy of *Cordyceps militaris* on the proliferation of B420, we performed an in vitro culture and observed no significant difference in the OD_600 nm_ in *Cordyceps militaris*-supplemented culture medium compared to controls, whether carbohydrates were supplemented or not (Figure 6C). Thus, *Cordyceps militaris* revealed no significant influence on the proliferation of B420. The reductions in the relative abundance of *Bifidobacterium* and copies of B420 were not attributable to the inhibition of *Cordyceps militaris* against B420. It has been reported that the gut microbiota ecology significantly influences probiotic proliferation through factors such as competition for resources, the production of inhibitory metabolites, and the pre-existing environment [32]. The resident microbes, especially *Cordyceps militaris*-promoting *A. muciniphila*, may be attributable to the inhibition against B420 after treatment with *Cordyceps militaris*.

Even though the copies of B420 in the feces were significantly decreased by *Cordyceps militaris*, the synergistic effect was still observed. Therefore, we hypothesized that the dead B420 could collaborate with *Cordyceps militaris* to produce the synergistic effect. HFD-fed mice were supplemented with *Cordyceps militaris* or heat-killed B420 and *Cordyceps militaris* for 8 weeks. However, no appreciable variation in weight increase was detected (Figure 6D), indicating that live B420, rather than dead B420, collaborated with *Cordyceps militaris* to produce the synergistic effect.

Next, we investigated the effects of metabolites of live B420 and *Cordyceps militaris* incubated with gut microbiota in feces from HFD-fed mice on the proliferation and mucin 2 expression of HT29-MTX-E12 cells, goblet cell-like subclones sourced from the human colorectal adenocarcinoma cell line HT29 via selection with methotrexate. The metabolites of live B420 and *Cordyceps militaris* collaboratively reversed the negative effect of the HFD on the survival of HT29-MTX-E12 cells (Figure 6E). A synergistic effect was also observed for the mRNA expression of mucin 2 (Figure 6F). The effects of metabolites of live B420 and *Cordyceps militaris* on the proliferation and mucin 2 expression in HT29-MTX-E12 cells were affirmed by staining with Alcian blue (Figure 6G). In a human clinical trial, a symbiotic product incorporating B420 and polydextrose was found to synergistically increase the relative proportion of *Akkermansia* spp. in the human fecal microbiota [7]. The polysaccharides of *Cordyceps militaris* were complex, as their structures varied with extraction methods [33]. None of the fundamental structural features of some polysaccharides extracted from *Cordyceps militaris* were polydextrose. However, the prevalence of *A. muciniphila* in the intestinal tract was reported to be obviously promoted by polysaccharides of *Cordyceps militaris* [16,34]. Overall, these results support that the metabolites of live B420 and *Cordyceps militaris*-derived metabolites of the gut microbiota mediated the increased production of mucin 2.

## 4. Conclusions

This study illustrates that B420 and *Cordyceps militaris* synergistically prevented obesity by collaboratively promoting the production of mucin 2 in the colon and a profound shift in obesity-related gut microbiota, induced by the bloom of commensal *A. muciniphila*. While further exploration is required to explicate the exact metabolites mediating these effects, this work provides novel insights and strategies for the enhanced therapeutic effects of probiotics and medicine food homology against obesity.

## Data Availability

The data that support the findings of this study are available from the corresponding author upon reasonable request due to privacy.

## Supplementary Materials

The following supporting information can be downloaded at: https://www.mdpi.com/article/10.3390/nu18081195/s1, Table S1: Primer sequences; Table S2: Composition of the Medium.
