# Supplementary material for: Bifidobacterium animalis ssp. lactis 420 and Cordyceps militaris Synergistically Modulate the Gut Microbiota by Increasing Mucin 2 Production
Source: Nutrients. 2026 Apr 10;18(8):1195. doi: 10.3390/nu18081195 (PMC13118426; doi:10.3390/nu18081195)
Supplement: Supplementary file 1 [file nutrients-18-01195-s001.zip › nutrients-4204904-supplementary.pdf]

Bifidobacterium animalis ssp. lactis 420 **and** ***Cordyceps militaris*** **synergistically modulate the gut microbiota by increasing mucin 2 production**

Ziyang Deng[a,c], Yi Wang[a], Jike Shuai[a,c], Miaomiao Chen[b], Shuai Yang[b], Donghong Liu[a,c], Xingqian Ye[a,d], Shiguo Chen [a,c,*], Haibo Pan[a,c,*],

[a]*College of Biosystems Engineering and Food Science, Zhejiang University, Hangzhou 310058, China*

[b] *Tonicare Electronic Commerce (Hangzhou) Co., Ltd., Hangzhou 310016, Zhejiang, China*

[c]*Innovation Center of Yangtze River Delta, Zhejiang University, Jiaxing 314102, China*

[d] *Zhejiang University Zhongyuan Institute, Zhengzhou 450000, Henan, China*

[*] Corresponding authors at: College of Biosystems Engineering and Food Science, Zhejiang University, 866 Yuhangtang Rd, Hangzhou, China.

Email addresses: apanhaibo@126.com (H. Pan)

College of Biosystems Engineering and Food Science, Zhejiang University, 866 Yuhangtang Rd, Hangzhou, China.

Email addresses: chenshiguo210@163.com (S. Chen)

1. Serum biochemical analysis

Biochemical analyses of serum were performed using an automatic 7020 biochemistry analyzer (Hitachi, Japan) according to the protocol provided by the manufacturer.

2. Liver triglyceride analysis

Liver triglyceride was measured with chloroform-methanol extraction and enzymatic reactions with a triglycerides assay kit (Thermo Fisher Scientific, China), according to the protocol provided by the manufacturer.

3. Endotoxin measurements

Serum endotoxin levels were measured using a recombinant factor c endotoxin detection kit (Lonza, China) according to the manufacturer's instructions.

4. Hematoxylin and Eosin (H&E) staining

Subsections of visceral fat, liver and colon were partially embedded in 10% neutral buffered formalin solution. The hepatic central lobe was collected and punched sections were fixed in 10% NBF. Paraffin embedded tissue sections (4 μm) were stained with H&E for morphological examination.

5. Oil Red O Staining

Livers were embedded and sectioned at 4 mm thickness and stained with Oil red O for 6 min. After staining, the sections were washed using running tap water, and mounted using a sterile glycerol solution.

6. Fecal microbiota DNA extraction

In detail, fecal microbiota DNA was extracted using the QIAamp DNA Stool

Mini Kit (Qiagen, USA). Tubes containing specimen and steel bead were placed into a benchtop homogenizer and disrupted for 30 s three times, with a 1-min rest period. Microbiota samples were collected by period. Microbiota samples were collected by low-speed centrifugation and high-speed centrifugation after homogenization, and further treated as described in the speed centrifugation after homogenization, and further treated as described in the manufacturer's instructions.

7. Illumina HiSeq sequencing and library construction

Fecal samples were snap frozen in liquid nitrogen and stored at 80°C. DNA was extracted using a fecal DNA isolation kit (Qiagen). 16S rDNA gene comprising the V3-V4 regions was amplified using composite primers containing a unique 10 base barcode to tag PCR products. PCR mix (50 μL) contained 25 ng DNA template, 5× HiFi buffer, 10 mM dNTP mix, 1 unit/μl HiFi DNA polymerase (KAPA Biosystems, USA) and 0.3 μM of composite primer pairs. PCR reaction conditions consisted of denaturation at 95 °C for 3 min, followed by 15-25 cycles of 98°C for 20 s, 45 °C for 15 s, and 72 °C for 15 s, and a final extension of 72 °C for 1 min. Replicate PCRs were pooled and amplicons were purified using the QiaQuick PCR Purification Kit (Qiagen). PCR amplicons were sequenced using the Illumina sequencing platform following the instructions of the manufacturer and HiSeq procedures. Sequencing libraries were generated using the TruSeq DNA PCR Free Sample Preparation Kit (Illumina, USA) following the manufacturer's recommendations. Library quality was assessed using the Qubit@ 2.0 Fluorometer (Thermo Scientific) and Agilent Bioanalyzer 2100 system. The library was sequenced on an Illumina HiSeq 2500 platform and 250 bp paired end

reads were generated.

8. 16S rDNA gene-based gut microbial analysis

Generated and demultiplexed sequences were analyzed using the QIIME software package (version 1.9.1). Paired-end sequences were merged with at least a 50-bp overlap. Resulting sequences containing ambiguous or low quality reads (Phred score $\leqslant$ 25) were removed from the dataset. Forward and reverse primers were trimmed from the filtered sequences; reads with at least one reverse primer mismatch or where the reverse primer was not found were discarded. Chimera checking and filtering was performed using UCHIME. Operational Taxonomic Units (OTU) picked from post-filtering reads were performed using USEARCH 61 version 6.1.544 with an open-reference methodology, which consisted of clustering sequences *de novo* at 97% identity threshold if they did not hit the reference sequence collection. Representative OTU sequences were assigned taxonomy against the Greengenes reference database using the RDP-classifier. Singleton OTUs and OTUs with a number of sequences < 0.005% of total number of sequences were discarded at this step.

9. Diet composition

The chow diet (AIN-93M, Research Diets) contained 14% protein, 73% carbohydrate, and 4% fat by weight (3.6 kcal/g). The high-fat diet (D12492, Research Diets) contained 26% protein, 26% carbohydrate, and 35% fat by weight (5.24 kcal/g), with 60% of calories derived from fat (lard-based).

Table S1: Primer sequences

Target gene	Direction	Direction Primer sequence (5’-3’)
zonula occludens-1	Forward	ACCCGAAACTGATGCTGTGGATAG
	Reverse	AAATGGCCGGGCAGAACTTGTGTA
claudin-1	Forward	CCCGGAAAACAACCTC
	Reverse	TTTGCTCCAGGAAGATCTC
Mucin 2 (mouse)	Forward	GCTGACGAGTGGTTGGTGAATG
	Reverse	GATGAGGTGGCAGACAGGAGAC
Mucin 2 (human)	Forward	ACTCTCCACACCCAGCATCATC
	Reverse	GTGTCTCCGTATGTGCCGTTGT
glyceraldehyde-3-phosphate dehydrogenase	Forward	GCATCCACTGGTGCTGCC
	Reverse	TCATCATACTTGGCAGGTTTC
Akkermansia muciniphila	Forward	CAG CAC GTG AAG GTG GGG AC
	Reverse	CCT TGC GGT TGG CTT CAG AT
Bifidobacterium animalis ssp. lactis 420	Forward	ACCAACCTGCCCTGTGCACCG
	Reverse	CCATCACCCCGCCAACAAGCT

Table S2: Composition of the Medium

Culture Medium	Ingredients	Concentration (g/L)
Brain Heart Infusion	Calf Brain Infusion	7.7
	Beef Heart Infusion	9.8
	Peptone	10
	Glucose	2
	Sodium Chloride	5
	Disodium Phosphate	2.5
